# LSTrAP: efficiently combining RNA sequencing data into co-expression networks

**DOI:** 10.1186/s12859-017-1861-z

**Published:** 2017-10-10

**Authors:** Sebastian Proost, Agnieszka Krawczyk, Marek Mutwil

**Affiliations:** Max-Planck Institute for Molecular Plant Physiology, Am Muehlenberg 1, 14476 Potsdam, Germany

**Keywords:** Transcriptomics, Co-expression, RNA-Seq analysis, Large-scale biology, Network analysis, Gene function prediction, Expression atlas

## Abstract

**Background:**

Since experimental elucidation of gene function is often laborious, various in silico methods have been developed to predict gene function of uncharacterized genes. Since functionally related genes are often expressed in the same tissues, conditions and developmental stages (co-expressed), functional annotation of characterized genes can be transferred to co-expressed genes lacking annotation. With genome-wide expression data available, the construction of co-expression networks, where genes are nodes and edges connect significantly co-expressed genes, provides unprecedented opportunities to predict gene function. However, the construction of such networks requires large volumes of high-quality data, multiple processing steps and a considerable amount of computation power. While efficient tools exist to process RNA-Seq data, pipelines which combine them to construct co-expression networks efficiently are currently lacking.

**Results:**

LSTrAP (Large-Scale Transcriptome Analysis Pipeline), presented here, combines all essential tools to construct co-expression networks based on RNA-Seq data into a single, efficient workflow. By supporting parallel computing on computer cluster infrastructure, processing hundreds of samples becomes feasible as shown here for *Arabidopsis thaliana* and *Sorghum bicolor*, which comprised 876 and 215 samples respectively. The former was used here to show how the quality control, included in LSTrAP, can detect spurious or low-quality samples. The latter was used to show how co-expression networks are able to group known photosynthesis genes and imply a role in this process of several, currently uncharacterized, genes.

**Conclusions:**

LSTrAP combines the most popular and performant methods to construct co-expression networks from RNA-Seq data into a single workflow. This allows large amounts of expression data, required to construct co-expression networks, to be processed efficiently and consistently across hundreds of samples. LSTrAP is implemented in Python 3.4 (or higher) and available under MIT license from https://github.molgen.mpg.de/proost/LSTrAP

**Electronic supplementary material:**

The online version of this article (10.1186/s12859-017-1861-z) contains supplementary material, which is available to authorized users.

## Background

Experimentally determining a gene’s function is laborious and time consuming, therefore numerous in silico methods have emerged to predict gene function [1]. Some methods assign functions based on sequence similarity to known domains [2] or genes with a known function [3], or matching protein structure to known templates [4]. Furthermore, genes whose protein products physically interact can be implicated to be part of the same biological process [5]. Expression patterns across various tissues, developmental stages and conditions can shed light on when and where a gene is required, which in turn provides clues about the gene’s function. To this end, numerous platforms emerged that allow browsing such expression profiles (e.g. eFP browser [6], Genevestigator [7] and PaGenBase [8]). Genes involved in the same biological process are often transcriptionally coordinated. Such co-expression relationships can be represented as networks [9], which allow the function of characterized genes to be transferred to uncharacterized neighbours in the network. This principle has successfully been used to predict gene function in various species from various kingdoms [10, 11]. Finally, integrative methods have been developed that leverage multiple types of evidence to detect functionally coherent modules or co-function networks [12, 13].

Expression based methods have been especially powerful as, since the onset of microarrays, they can be used to simultaneously measure expression levels of thousands of genes. However, expression of a substantial fraction (up to 40% for popular microarray platforms [10]) of genes might not be captured by the microarrays, due to absence of probes interrogating these genes. RNA sequencing (RNA-seq) which does allow determining expression levels of a near-complete set of genes has become the norm, as RNA-Seq has become increasingly affordable.

To construct co-expression networks, a sufficiently large set of different tissues, developmental stages, and biotic/abiotic perturbations needs to be collected at a sufficient read depth. While the bare minimum was estimated to be >20 samples with >10 million reads, more samples and higher read depth were found to increase the predictive capabilities of the resulting network [14]. Currently, the number of RNA-Seq experiments in the Sequence Read Archive (SRA) [15] is growing rapidly and constructing RNA-Seq based co-expression networks has become feasible for various species [16–18].

While constructing expression atlases and co-expression networks can facilitate gene function prediction, it poses multiple challenges. First, after collecting and annotating data, several tools (e.g. Trimmomatic [19], Bowtie 2 [20] and TopHat2 [21]) need to be run consistently across all samples. Furthermore, as processing large quantities of RNA-Seq data requires a substantial amount of computational power, parallelization of jobs on a computer cluster is imperative for keeping runtimes within reasonable limits. Finally, as the construction of co-expression networks often relies on publicly available data from various different sources, quality metrics to detect potential problems with RNA-seq samples need to be implemented.

Co-expression networks, once established for multiple species, have been combined with functional and comparative genomics [22]. For instance the comparison of co-expression networks from tomato and potato has led to the discovery of gene modules associated with steroidal glycoalkaloids [23]. Furthermore, the merger of co-expression networks with phylogenetic data revealed how gene modules relevant for cell wall synthesis evolved independently in mosses and land plants [24].

To address these challenges, we present the Large-Scale Transcriptome Analysis Pipeline (LSTrAP), which pre-processes RNA-seq data, maps it to the genome, performs quality control and produces co-expression networks, along with (optionally) functional and comparative genomics data to enable a host of downstream analyses. A manual is included for users outlining steps to configure the pipeline on their system for their data. Furthermore, several additional scripts are included to assist users to obtain pre- and post-process results. The output from LSTrAP is compatible with third party applications such as Cytoscape [25] to mine networks for novel biological information.

## Implementation

**Fig. 1:**
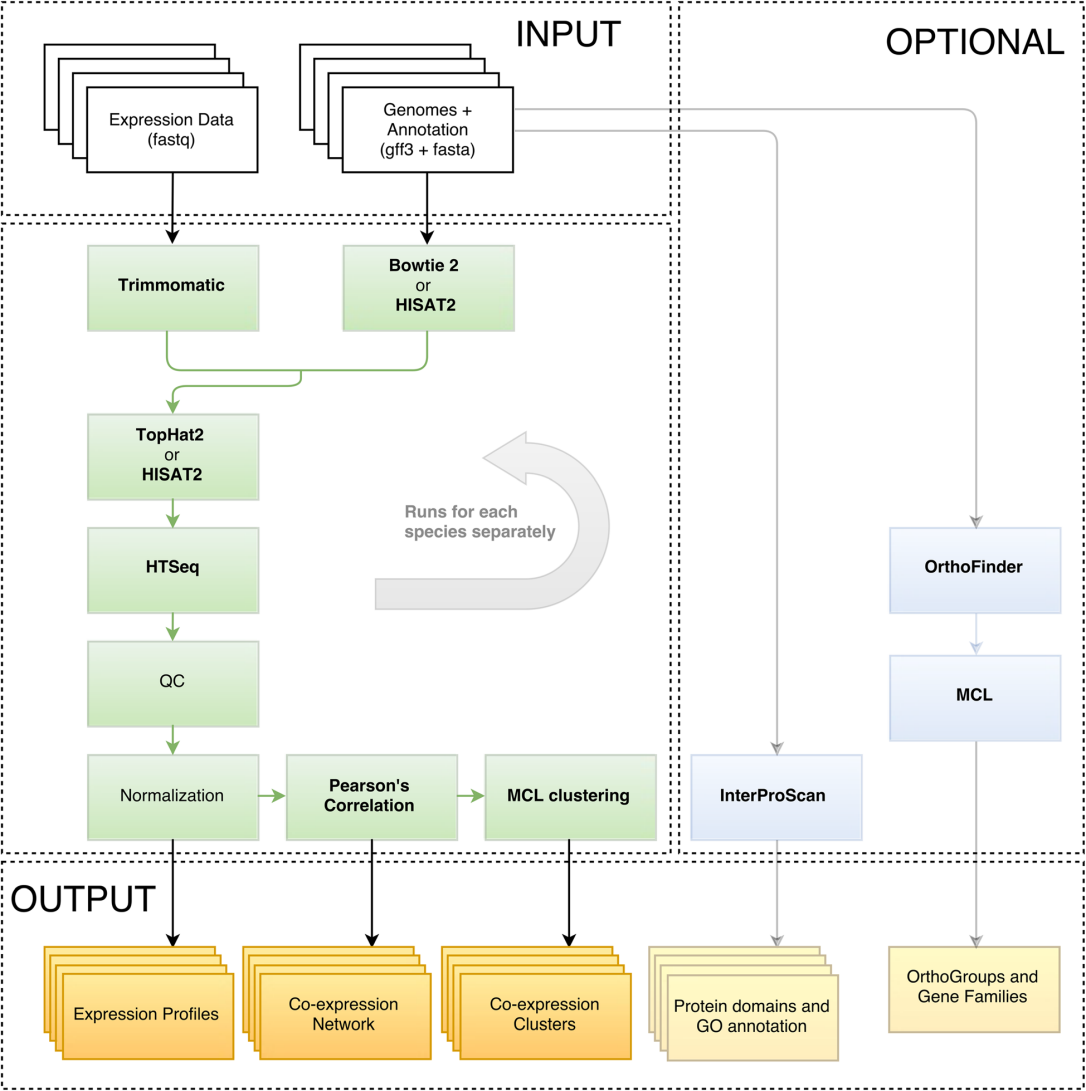
LSTrAP overview. All tools to process RNA-Seq data (green boxes) along with optional protein domain annotation and detection of orthologs and gene families (blue boxes) are combined into a single workflow

LSTrAP runs all required steps to construct co-expression networks for multiple species from raw RNA-Seq expression data, using a single command. This includes read-trimming, adapter cutting, read mapping, generation of normalized expression profiles, the construction of co-expression networks and the detection of co-expression clusters (Fig. 1). The pipeline iterates over all species and executes all steps that can be run in parallel as jobs on a computer cluster (default Oracle Grid Engine (previously Sun Grid Engine [26]),with support for PBS [27] / Torque [28]). Additionally, included quality control metrics indicate which RNA-seq samples are potentially unsuited or of low quality.

### Data acquisition

De-multiplexed RNA-seq data should be provided to LSTrAP as (compressed) fastq files in one directory per species. Publicly available expression data stored in the Sequence Read Archive (SRA) [15] can be downloaded in bulk using the Aspera download client and converted to fastq format with *get_sra_ip.py* and *sra_to_fastq.py* scripts, respectively (found in LSTrAP repository). Data provided in other formats (such as BAM files) needs to be converted to fastq using e.g. SAMTools [29], BEDTools [30] or Picard (http://broadinstitute.github.io/picard).

Apart from expression data, LSTRaP requires the genome sequence in fasta format together with a gff3 file describing where in the genome coding sequences are located. Alternatively, a fasta file with coding sequences can also be used and gff3 file can be generated with helper script *fasta_to_gff.py*.

Note that for species with multiple splice variants, it is recommended to keep either the primary or the longest transcript representative for a given gene, as HTSeq-Count [31], and therefore by extension LSTrAP, only considers reads that unambiguously map to a single gene model. Including splice variants will result in loss of reads that map to shared parts of isoforms. Including multiple splicing isoforms would reject reads that map to more than one gene model. The script *parse_gff.py* in the helper directory can be used to extract the longest splice variant from gff3 files. Additionally, to identify gene families and group orthologs using OrthoFinder [32], a fasta file with coding sequences for each transcript and a file with the resulting proteins is required. Here, the fasta header should contain only the gene identifier and match the gene identifiers used in the gff3 file.

### Preparing to run LSTrAP

To start the pipeline, two INI files need to be provided, one describing paths to third party tools (such as Trimmomatic [19] and TopHat2 [21]) and another specifying where to find the input data and desired output paths (example files and detailed instructions are provided in the documentation). Prior to starting the pipeline, LSTrAP will inform the user of any missing fields or paths. Once the input data and INI files are ready, LSTrAP can be started. Additional parameters to skip optional parts, resume from or stop at a given point are available. All the steps are executed in order and without further manual intervention.

### Running LSTRaP

#### Indexing the genome

For efficient mapping using TopHat2 [21] or HISAT2 [33], LSTrAP first creates a genome index file using Bowtie 2 [20] or hisat2-build. Default parameters are used to run Bowtie 2/hisat2-build, though different parameters can be provided through the configuration INI file.

By default BowTie 2 and TopHat2 are used by LSTrAP, add the parameter -use-hisat2 when running LSTrAP to switch to HISAT2.

#### Quality trimming and adapter cutting

As data might originate from different labs, generated using various protocols and sequenced using different platforms, quality trimming is included to ensure all samples adhere to the same minimal standard. To this end, fastq files are first processed using Trimmomatic [19] to ensure low quality bases are trimmed off. Furthermore, Trimmomatic can remove residual adapter sequences (a file with potential adapters needs to be specified, adapter-sequences for commonly used TruSeq Kits are included). The desired settings for Trimmomatic (minimal required read length, quality trimming parameters) can be set in the config INI file.

#### Read mapping

Trimmed reads are mapped to the indexed genome using TopHat2 [21] or HISAT2 [33], which will create BAM or SAM files containing the alignment of each read with regions in the genome (or transcriptome). The number of cores TopHat2/HISAT2 can use to process a single sample, along with other parameters can be specified in the config INI. By default LSTrAP will start TopHat2/HISAT2 using 4 cores and standard settings.

#### Gene expression and normalization

For each gene (as defined by the gff file), the number of reads mapping uniquely to that gene are counted. To this end, HTSeq-Count [31] is included in the pipeline, which produces for each sample a file containing the mapped reads per gene. LSTrAP aggregates those files into a single (*m* x *n*) matrix containing the expression value for each gene (*m*) in each sample (*n*). Normalization for differences in sequencing depth between samples, and gene length is required. LSTrAP will normalize the expression matrix using two commonly used approaches; Transcripts Per Kilobase per Million (TPM) and Reads Per Kilobase per Million (RPKM) [34].

#### Co-expression networks and clusters

LSTrAP includes a fast implementation, using NumPy’s [35] matrix operations, to calculate Pearson correlation coefficients (PCC), which has been found to be among the most performant for RNA-Seq based co-expression studies [14], based on the TPM normalized expression matrix. The PCC value ranges from −1.0 to 1.0 where zero means no correlation, positive values indicate various degrees of correlation (1.0 being perfectly correlated) and negative values correspond with anti-correlation (−1.0 would be perfect anti-correlation). The result is a table describing for each gene the 1000 strongest co-expressed genes in the dataset. All pairs with a PCC value >0.7 (the recommended setting when using MCL on co-expression data) are stored separately and represent the global co-expression network, which is clustered into groups of co-expressed genes using the MCL algorithm [36]. Note that depending on the intended use-case, applying additional, more stringent filters can provide better results.

#### Functional and comparative features

To facilitate further functional studies, LSTRaP includes InterProScan [2] as an optional part of the pipeline (Fig. 1, blue boxes). InterProScan will compare a gene’s protein product against a large database of known protein domains, and report regions in the protein that match entries in the database. Furthermore, Gene Ontology terms (GO) associated with domains are assigned to genes as well.

To enable comparative studies (e.g. Movahedi et al. [37] and Ruprecht et al. [24]), orthologous genes (genes derived through speciation events) are detected using OrthoFinder [32]. Gene families (genes derived from a common ancestor) are generated by using MCL [36] directly on OrthoFinder’s BLAST output.

## Results

### Quality control

Unsuited or low-quality expression data can negatively affect the final co-expression network [38]. To avoid inclusion of such samples, LSTrAP indicates which samples are potentially problematic based on two metrics; the percentage of reads TopHat2 [21] (or HISAT2 [33]) is able to map to the genome and the fraction of those reads that HTSeq-Count maps to coding sequences. For example, samples from one species should map poorly to the genome from another (low % of mapped reads reported by TopHat2/HISAT2), while DNA sequencing samples should map less to coding sequences than polyA-enriched samples (low % of mapped reads reported by HTSeq-Count).

To investigate if these metrics can discriminate suited from unsuited samples, manually curated sets of positive and negative samples were processed and compared. As the positive dataset, 821 polyA-enriched, annotated RNA-seq samples for *Arabidopsis thaliana*, were selected from SRA archive. The negative dataset comprised 36 samples, (Additional file 1: Table S1, Additional file 1: Methods S1). These include DNA-seq samples, other species than *Arabidopsis thaliana*, non-polyA-enriched samples, samples derived from DNA and different ecotypes of *Arabidopsis thaliana*.

**Fig. 2:**
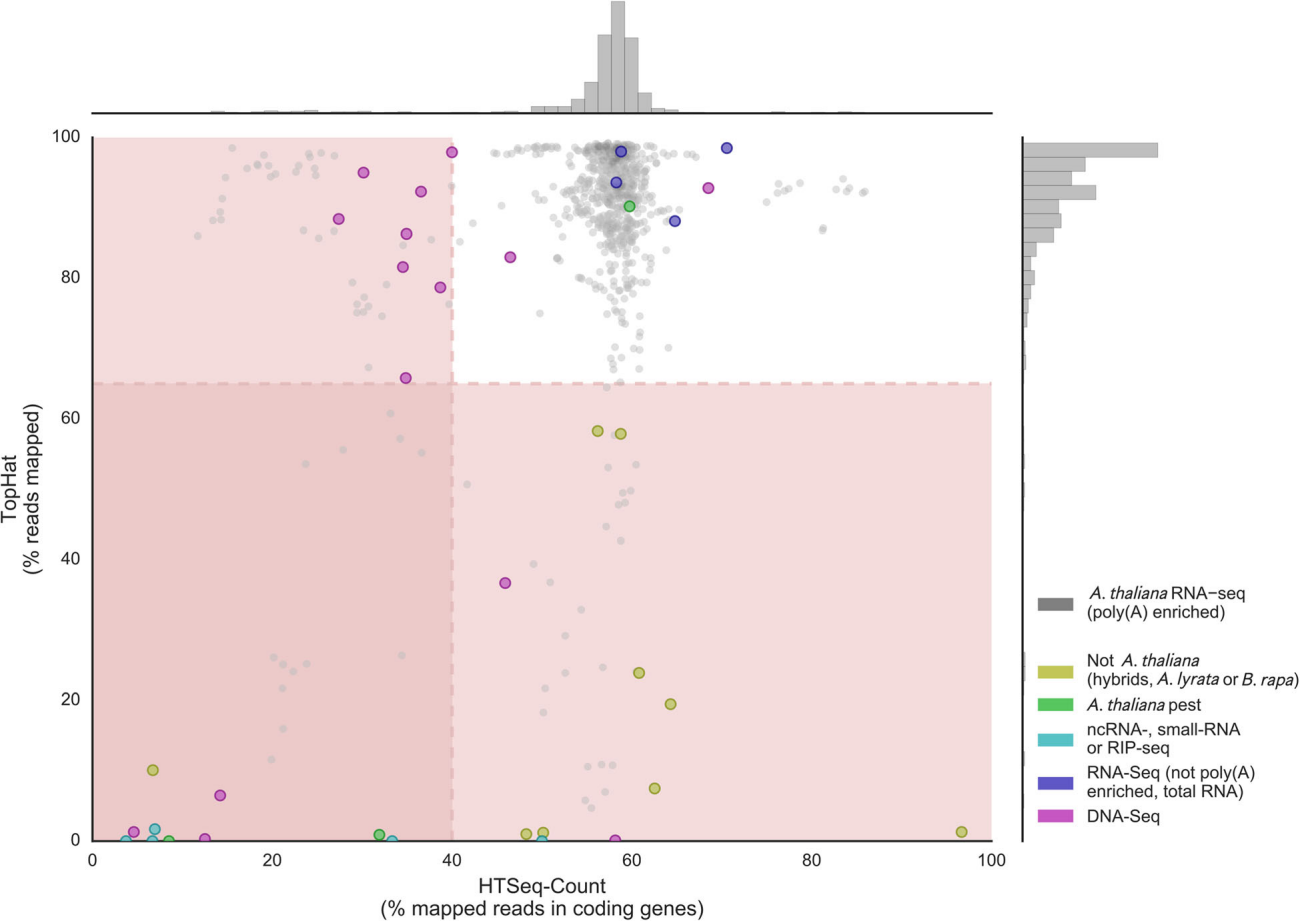
TopHat’s % reads mapped and HTSeq-count’s (% of reads mapped onto coding genes) for *Arabidopsis thaliana*. Gray dots indicate samples included in the positive dataset, containing only *Arabidopsis thaliana*, poly(A) enriched samples. Other samples, generally considered un-suited for the construction of co-expression networks are indicated by other colors. Samples derived from related organisms are shown in yellow, while samples from pests infecting *Arabidopsis thaliana* are shown in green. *Arabidopsis thaliana* samples from DNA-seq derived samples are shown in purple while non-poly(A) enriched samples are shown in cyan (samples enriched for various types of non-coding RNA) or blue (whole RNA extracts). The exact mapping values are provided in Additional file 1: Table S1. LSTrAP by default warns users if samples with low mapping statistics are included (red areas in graph, HTSeq-Count <40% or TopHat <65%)

We observed that the majority of the samples from the positive dataset, which contains poly(A) enriched RNA-Seq samples, (Fig. 2, gray points) have a higher fraction of reads mapping to the genome and coding sequences, compared to samples in the negative dataset (Fig. 2, Additional file 1: Table S1). This indicates that for valid *Arabidopsis thaliana* samples, over 65% of reads should map to the genome and, of these, at least 40% of those reads should map to coding sequences (Fig. 2, samples outside red areas). Reads from samples from unwanted species map poorly to the Arabidopsis genome (yellow and green dots), with one notable exception: SRR1695529, Mycorrhiza infecting *Arabidopsis thaliana*. This sample contained parts of *Arabidopsis thaliana* roots, and thus a substantial amount of *Arabidopsis thaliana* RNA, as well [39]. Studies, which sequenced only ncRNA and small RNAs (cyan dots), map poorly to the genome. While relatively few reads from DNA derived samples (e.g. WGS and CHiP seq. Purple dots) map to coding genes. Samples which are not poly(A) enriched (blue dots) cannot be distinguished from poly(A) enriched samples using these metrics. Note that, samples that do not fulfil these criteria are still processed. To avoid falsely excluding biologically meaningful samples, the final decision whether those samples should be used to construct the co-expression network is left to the user. The suggested cutoffs can be altered in your data INI file.

### Construction of a co-expression network for *Sorghum bicolor*

To exemplify we included the following use case; poly(A) enriched RNA-seq runs for *Sorghum bicolor* were downloaded from the SRA [15] (215 samples in total), and manually determined the tissue type (e.g. root, leaf, flower) from each experiment’s description (Additional file 1: Method S1, Additional file 1: Table S2). All samples, along with Sorghum’s genome sequence [40], were processed using LSTrAP using default settings. Trimmomatic [19], with a minimum read length of set to 36 (the default in LSTrAP), excluded 11 runs since all reads in these runs were shorter than 36 bases, leaving 204 samples. An additional 17 samples were of insufficient quality based on the mapping statistics (Additional file 1: Figure S1), and were therefore excluded from the final network. The resulting co-expression network and TPM normalized expression matrix were used for further analysis.

**Fig. 3:**
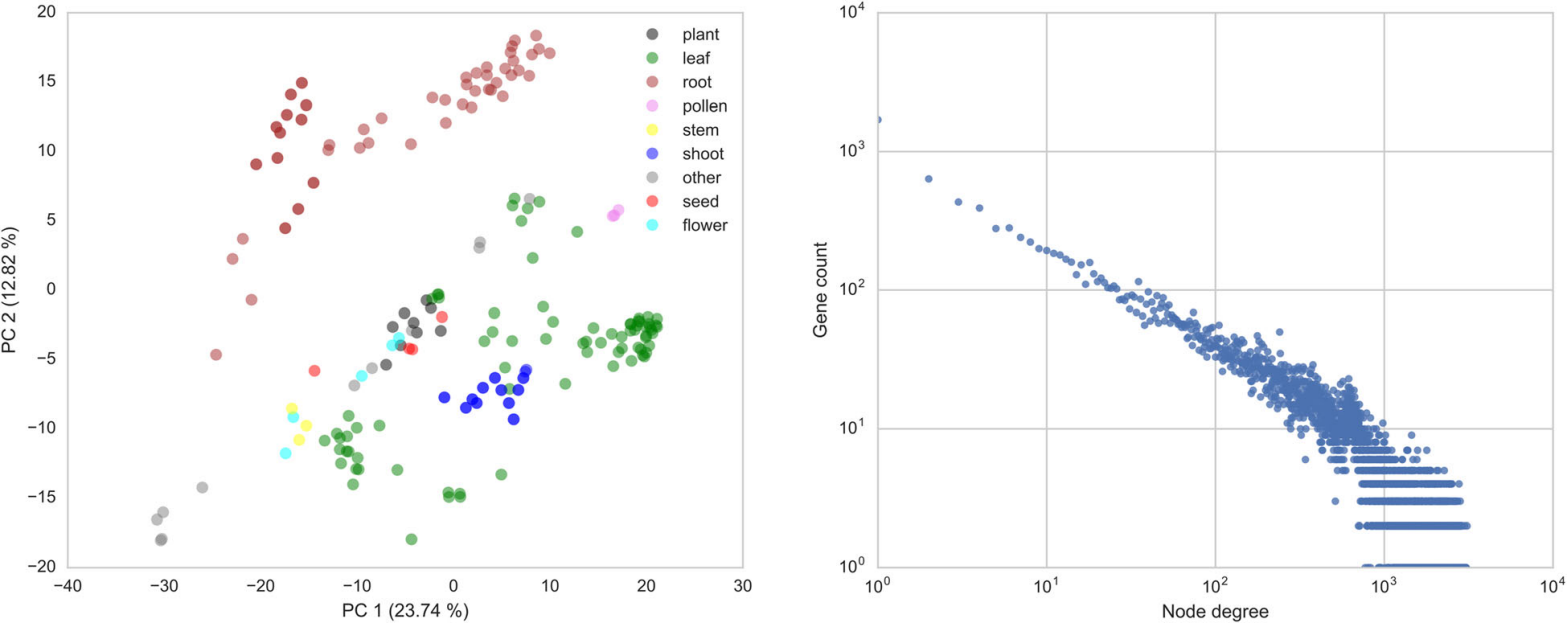
PCA analysis and node degree distribution of the *Sorghum bicolor* samples and co-expression network, respectively. **a** Principal Component Analysis of all Sorghum samples color coded by the tissue sampled. **b** Power law plot of the PCC > 0.7 co-expression network, where node degree and node frequency are shown on the x- and y-axis, respectively

First, a principal component analysis (PCA) of the expression matrix (Additional file 1: Method S2, Fig. 3a) revealed a clear separation of photosynthetic and non-photosynthetic tissues. As expected, samples derived from the same tissue tend to end up in proximity of each other in the plot. Furthermore, replicates cluster together (darker colors indicate overlapping nodes) and no outliers, which would indicate potential problems, were present. Alternatively, a hierarchically clustered heatmap (Additional file 1: Figure S2) can be generated to show relations between samples and detect potential outliers.

Biological networks, including co-expression networks, often follow a power-law behavior (also called scale-free or small worlds networks), where few nodes have many connections (so called hubs in the network) and many nodes have few connections [41, 42]. We plotted the frequency (number of genes) of the node degree (number of connections a node has) for the Sorghum network, considering only edges with PCC > 0.7 (Additional file 1: Method S3). Similarly to the co-expression network of *A. thaliana* (Additional file 1: Figure S3), the Sorghum co-expression network also follows power-law behavior, indicated by the points in Fig. 3b forming a straight line. Hence the co-expression network has the expected topology.

**Fig. 4:**
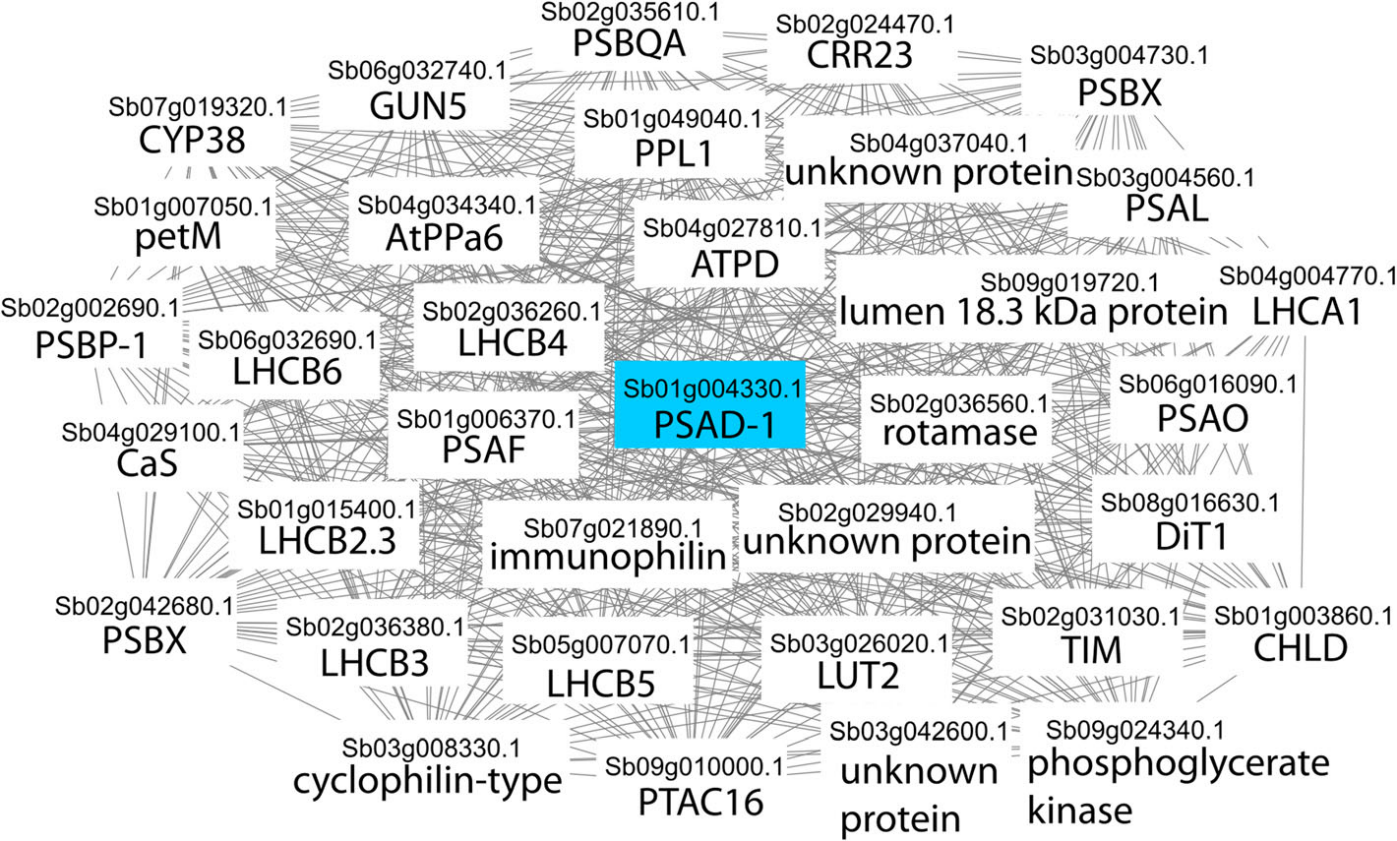
Co-expression neighborhood for Sb01g004330.1 (blue node). Nodes represent genes, while edges connect co-expressed genes. To easily visualize the neighborhood, we have decreased the number of genes co-expressed Sb01g004330.1 by increasing the PCC threshold to >0.925

Finally, to investigate whether the co-expression network can capture biologically meaningful information, we have investigated co-expression neighbors of Sb01g004330.1 PSAD-1 (Fig. 4), a photosystem I subunit required for photosynthesis. Directly connected to this gene are several other known components of Sorghum’s photosynthetic apparatus, but also three uncharacterized genes. The latter would be excellent candidates for future studies, as based on their position in the network they are likely involved in photosynthesis as well.

## Discussion

LSTrAP offers a single command solution to process a large volume of RNA-Seq samples and construct co-expression networks. Researchers working on species for which no co-expression networks exist, can construct one based on publically available data, similarly to the case study presented here on *Sorghum bicolor*. These co-expression networks can predict gene function, and thus help to identify relevant candidate genes in biological processes of interest and guide future experiments.

Smaller datasets, e.g. sampling a wild-type & mutant or control & treatment, which are insufficient to construct a co-expression network, can be prepared efficiently in LSTrAP for detection of differentially expressed genes (DEG). Read trimming, mapping and counting are shared among the RNA-Seq analyses. Therefore, running the pipeline up-to-and-including HTSeq-Count provides a simple one-step solution to obtain processed RNA-Seq data compatible with methods allowing DEG detection, such as DESeq2 [43].

As RNA-Seq technology and tools continues to improve future releases of LSTrAP will focus on including new and better tools into this workflow. Furthermore, downstream steps could be added to facilitate users in their quests to explore the generated co-expression networks.

## Conclusions

Expression profiles and co-expression networks have been proven to be valuable tools to predict functions of uncharacterized genes. However, building these networks using thousands of RNA-Seq samples was impractical. LSTrAP allows quick processing and quality assessment of large multi-species datasets to produce biologically meaningful co-expression networks. By further integrating functional and comparative data, LSTrAP enables the study of co-expression networks in a broad evolutionary context.

## Additional file

Additional file 1: Figure S1. Quality statistics for *Sorghum bicolor* samples. Gray dots indicate quality statistics of the samples based on HTSeq-Count and TopHat. Samples below our suggested quality control (contained within red areas in plot) were excluded from the final network. **Figure S2.** Dendrogram and heatmap of *Sorghum bicolor* sample distances. The helper script matrix_heatmap.py calculates the Euclidean distance between samples and plots a hierarchically clustered heatmap of those sample distances. This can be used to detect outliers. Here the most divergent samples (in the top left) are valid pollen and seed samples which are known to have a unique transcriptional profile. **Figure S3.** Node degree distribution of the *Arabidopsis thaliana* samples co expression network. Co-expression networks are known to have few nodes with many connections to other genes and many genes with few connections. For the co expression network of *Arabidopsis thaliana* based on the positive samples, this behavior can clearly be observed. **Table S1.** Negative *Arabidopsis thaliana* dataset. The columns correspond to SRA run IDs for the samples, short description (description and type) and mapping percentages for TopHat and HTSeq-count. **Table S2.** *Sorghum bicolor* samples with organ annotation. Overview of all *Sorghum bicolor* samples used, organized by organ the samples were derived from. **Methods S1.** Data source and curation. **Methods S2.** PCA analysis of expression data. **Methods S3.** Power law. (DOCX 411 kb)

## Abbreviations

LSTrAP: Large-scale transcriptome analysis pipeline
PCA: Principal component analysis
PCC: Pearson correlation coefficients
RPKM: Reads per kilobase per million
SRA: Sequence read archive
TPM: Transcripts per kilobase per million
