# Supplementary material for: LSTrAP: efficiently combining RNA sequencing data into co-expression networks
Source: BMC Bioinformatics. 2017 Oct 10;18:444. doi: 10.1186/s12859-017-1861-z (PMC5634843; doi:10.1186/s12859-017-1861-z)
Supplement: Additional file 1: Figure S1. — Quality statistics for Sorghum bicolor samples. Gray dots indicate quality statistics of the samples based on HTSeq-Count and TopHat. Samples below our suggested quality control (contained within red areas in plot) were excluded from the final network. Figure S2. Dendrogram and heatmap of Sorghum bicolor sample distances. The helper script matrix_heatmap.py calculates the Euclidean distance between samples and plots a hierarchically clustered heatmap of those sample distances. This can be used to detect outliers. Here the most divergent samples (in the top left) are valid pollen and seed samples which are known to have a unique transcriptional profile. Figure S3. Node degree distribution of the Arabidopsis thaliana samples co expression network. Co-expression networks are known to have few nodes with many connections to other genes and many genes with few connections. For the co expression network of Arabidopsis thaliana based on the positive samples, this behavior can clearly be observed. Table S1. Negative Arabidopsis thaliana dataset. The columns correspond to SRA run IDs for the samples, short description (description and type) and mapping percentages for TopHat and HTSeq-count. Table S2. Sorghum bicolor samples with organ annotation. Overview of all Sorghum bicolor samples used, organized by organ the samples were derived from. Methods S1. Data source and curation. Methods S2. PCA analysis of expression data. Methods S3. Power law. (DOCX 411 kb) [file 12859_2017_1861_MOESM1_ESM.docx]

**LSTrAP: Efficiently combining expression data into co-expression networks**

**Supplementary material**

Authors: Sebastian Proost^1^, Agnieszka Krawczyk^1^, Marek Mutwil^1^

Affiliations:

^1^ Max-Planck Institute for Molecular Plant Physiology, Am Muehlenberg 1, 14476 Potsdam, Germany

**Supplemental Figures**


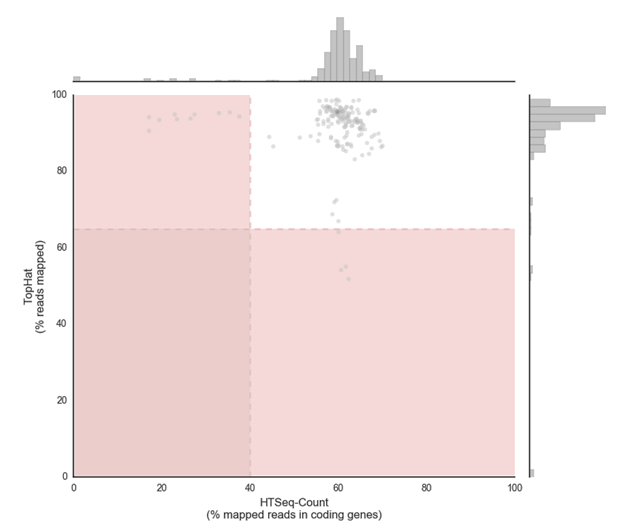

**Supplemental Figure 1. Quality statistics for *Sorghum bicolor* samples.** Gray dots indicate quality statistics of the samples based on HTSeq-Count and TopHat. Samples below our suggested quality control (contained within red areas in plot) were excluded from the final network.


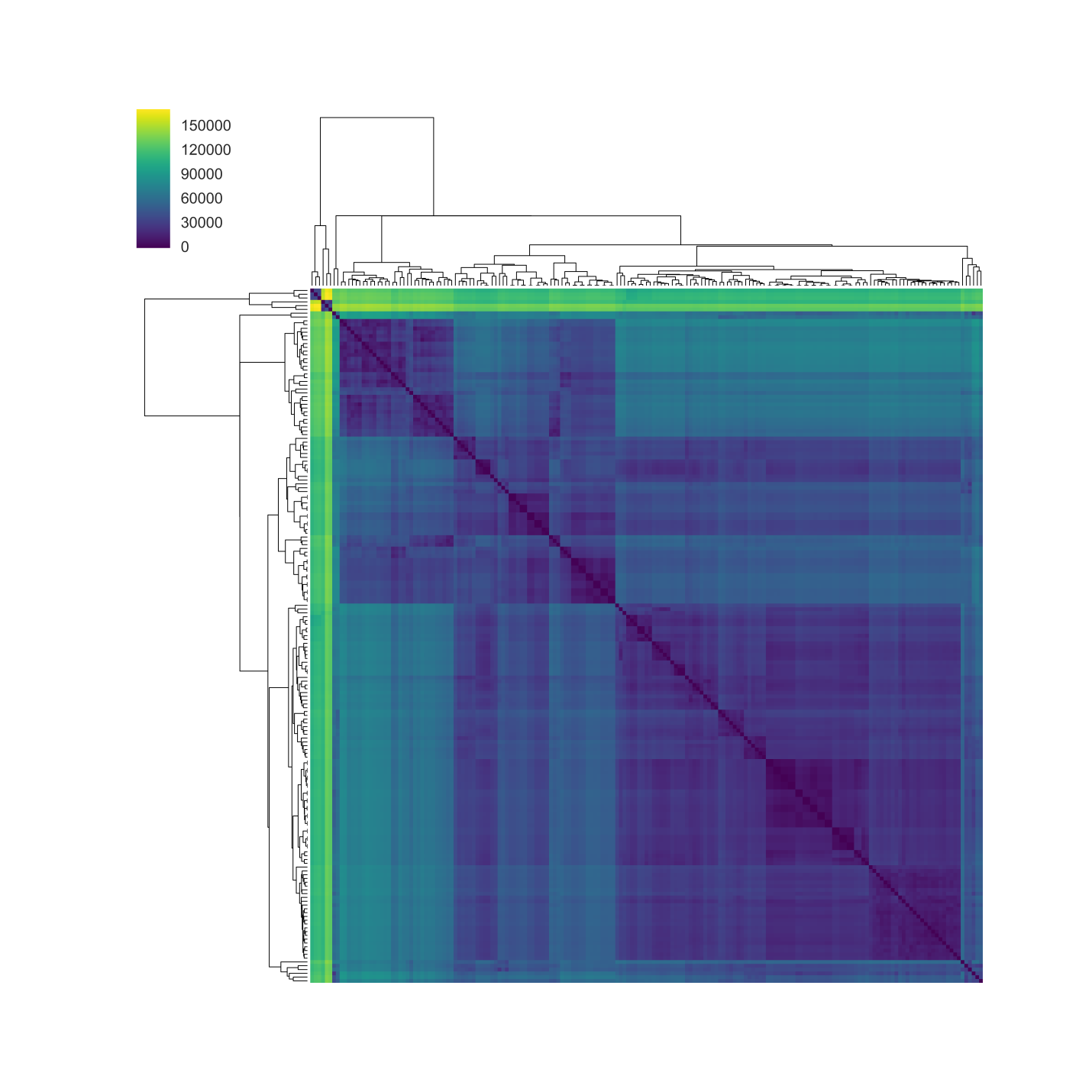


**Supplemental Figure 2. Dendrogram and heatmap of *Sorghum bicolor* sample distances.** The helper script *matrix_heatmap.py* calculates the Euclidean distance between samples and plots a hierarchically clustered heatmap of those sample distances. This can be used to detect outliers. Here the most divergent samples (in the top left) are valid pollen and seed samples which are known to have a unique transcriptional profile.

**
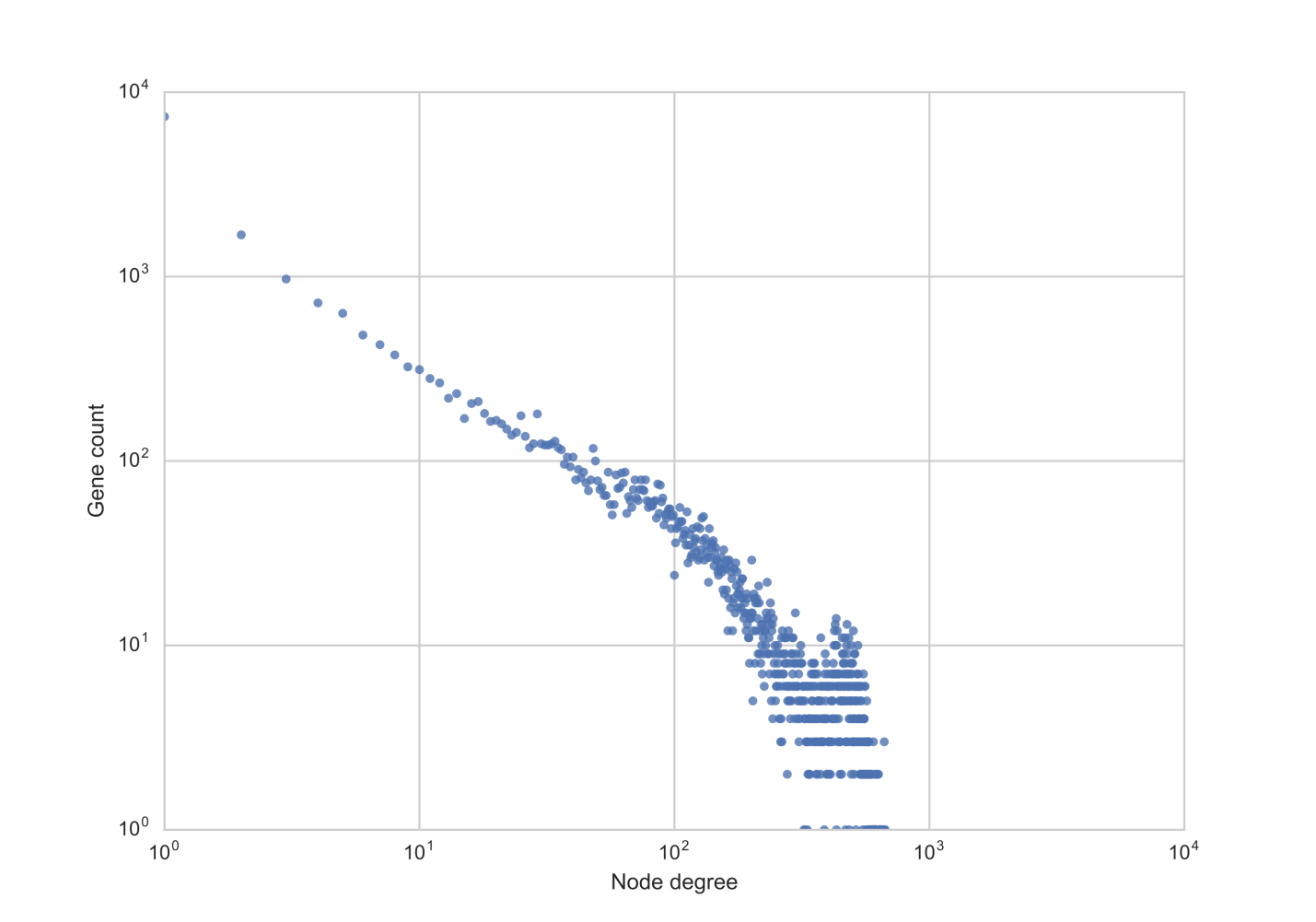
Supplemental Figure 3. Node degree distribution of the *Arabidopsis thaliana* samples co‑expression network**. Co-expression networks are known to have few nodes with many connections to other genes and many genes with few connections. For the co‑expression network of *Arabidopsis thaliana* based on the positive samples, this behavior can clearly be observed.

**Supplemental Tables**

|  |  |  | **TopHat** | **HTSeq-Count** |
| --- | --- | --- | --- | --- |
| **SRA run ID** | **Description** | **Type** | **% reads mapped** | **% mapped reads in cds** |
| SRR2142007 | *Arabidopsis thaliana*, ncRNA-Seq | Non-coding RNA-Seq | 1,7 | 6,9 |
| SRR2831380 | *Arabidopsis thaliana*, ncRNA-Seq | Non-coding RNA-Seq | <0,0001 | 3,7 |
| SRR1754057 | *Arabidopsis thaliana*, RIP-Seq | Non-coding RNA-Seq | <0,0001 | 50,0 |
| SRR1164433 | *Arabidopsis thaliana*, small RNA | Non-coding RNA-Seq | <0,0001 | 6,7 |
| SRR445214 | *Arabidopsis thaliana*, small RNA | Non-coding RNA-Seq | <0,0001 | 33,3 |
| ERR1447795 | *Arabidopsis thaliana*, total RNA | Not polyA-enriched RNA-Seq | 98,0 | 58,8 |
| SRR2049807 | *Arabidopsis thaliana*, total RNA | Not polyA-enriched RNA-Seq | 88,1 | 64,8 |
| SRR2079780 | *Arabidopsis thaliana*, total RNA | Not polyA-enriched RNA-Seq | 98,5 | 70,6 |
| SRR2993794 | *Arabidopsis thaliana*, total RNA | Not polyA-enriched RNA-Seq | 93,6 | 58,3 |
| ERR1368811 | *Arabidopsis lyrata*, RNA-Seq | Not *A. thaliana* | 23,9 | 60,8 |
| SRR2039799 | *Arabidopsis lyrata*, RNA-Seq | Not *A. thaliana* | 19,5 | 64,3 |
| SRR1232486 | *Arabidopsis thaliana x Arabidopsis arenosa*, RNA-Seq | Not *A. thaliana* | 1,3 | 96,7 |
| SRR3405446 | *Arabidopsis thaliana x Arabidopsis halleri*, RNA-Seq | Not *A. thaliana* | 57,9 | 58,8 |
| SRR3405437 | *Arabidopsis thaliana x Arabidopsis lyrata*, RNA-Seq | Not *A. thaliana* | 58,3 | 56,2 |
| DRR031147 | *Brassica rapa*, RNA-Seq | Not *A. thaliana* | 1,2 | 50,1 |
| SRR3189876 | *Brassica rapa*, RNA-Seq | Not *A. thaliana* | 10,1 | 6,7 |
| SRR3337089 | *Brassica rapa*, RNA-Seq | Not *A. thaliana* | 7,5 | 62,5 |
| SRR3584233 | *Brassica rapa*, RNA-Seq | Not *A. thaliana* | 1,0 | 48,3 |
| SRR2072471 | *Colletotrichum incanum*, infecting A.thaliana roots | Not *A. thaliana* | <0,0001 | 8,5 |
| SRR1695529 | *Serendipita vermifera*, mycorrhiza infecting *A.thaliana*, RNA-Seq | Not *A. thaliana* | 90,2 | 59,7 |
| SRR332280 | Spider mite feeding on *Arabidopsis thaliana*, RNA-Seq | Not *A. thaliana* | 0,9 | 31,9 |
| SRR1049788 | *Arabidopsis thaliana*, DNase I hypersensitivity | Not RNA-Seq | 92,8 | 68,5 |
| ERR903949 | *Arabidopsis thaliana*, bisulfite seq | Not RNA-Seq | 1,3 | 4,6 |
| SRR1138712 | *Arabidopsis thaliana*, bisulfite seq | Not RNA-Seq | 0,3 | 12,5 |
| SRR420836 | *Arabidopsis thaliana*, bisulfite seq | Not RNA-Seq | 6,5 | 14,2 |
| SRR2626466 | *Arabidopsis thaliana*, ChIP-Seq | Not RNA-Seq | 97,9 | 40,0 |
| SRR3040031 | *Arabidopsis thaliana*, ChIP-Seq | Not RNA-Seq | 92,3 | 36,5 |
| SRR3313886 | *Arabidopsis thaliana*, ChIP-Seq | Not RNA-Seq | 65,8 | 34,8 |
| SRR2926204 | *Arabidopsis thaliana*, DAP-Seq | Not RNA-Seq | 78,7 | 38,7 |
| SRR2926267 | *Arabidopsis thaliana*, DAP-Seq | Not RNA-Seq | 88,4 | 27,4 |
| SRR2926621 | *Arabidopsis thaliana*, DAP-Seq | Not RNA-Seq | 81,6 | 34,5 |
| SRR648299 | *Arabidopsis thaliana*, Exome | Not polyA-enriched RNA-Seq | 36,7 | 45,9 |
| DRR045409 | *Arabidopsis thaliana*, WGS | Not RNA-Seq | 86,3 | 34,9 |
| ERR1406037 | *Arabidopsis thaliana*, WGS | Not RNA-Seq | 95,0 | 30,1 |
| SRR3473160 | *Arabidopsis thaliana*, WGS | Not RNA-Seq | 83,0 | 46,5 |
| ERR1104211 | *Arabidopsis thaliana*, WGS + fungi and bacteria | Not RNA-Seq | 0,1 | 58,1 |

**Supplemental Table 1.** **Negative *Arabidopsis thaliana* dataset**. The columns correspond to SRA run IDs for the samples, short description (description and type) and mapping percentages for TopHat and HTSeq-count.

| **Tissue** | **SRA** |
| --- | --- |
| leaf | SRR1879518; SRR3061577; DRR001055; SRR1891234; SRR1832809; SRR1874061; DRR014992; DRR014989; DRR014985; SRR3063527; SRR1888240; SRR1832808; DRR014994; SRR1874038; SRR1874028; SRR1874030; SRR1823059; SRR1874033; DRR014988; SRR1874041; DRR006369; SRR1873792; DRR014986; SRR1878107; SRR1874049; DRR006372; SRR349643; SRR1874043; SRR1874036; SRR1822671; SRR1874035; DRR014990; DRR006371; SRR1889422; DRR014981; SRR1884878; SRR1829997; SRR1874031; SRR1874046; DRR014993; SRR3063529; DRR014984; SRR1890269; SRR1880959; SRR1882919; DRR006373; DRR014982; SRR1874047; DRR014979; SRR1832811; SRR1875520; SRR1881929; DRR014980; SRR1887380; SRR1874039; SRR1874040; SRR1822329; DRR014991; SRR1874037; SRR1874021; DRR014978; DRR014983; SRR1874023; DRR006370; SRR1873791; SRR3061391; SRR1874027; ERR886715; DRR001054; SRR1823060; ERR886716; SRR1874067; DRR001053; SRR1874042; SRR1883966; SRR1874026; SRR1876991; DRR014987; SRR1886389 |
| microspore | SRR3086642; SRR3086980; SRR3087078 |
| spikelet | DRR030762; DRR030761; DRR030763 |
| stem | DRR030765; DRR030764; DRR030766 |
| shoot | SRR299239; SRR959791; SRR299248; SRR959794; SRR959792; SRR299249; SRR299244; SRR299245; SRR299246; SRR299250; SRR959793; SRR299241; SRR299247; SRR299242; SRR299243; SRR959795; SRR959790; SRR299240; SRR959796; SRR959798; SRR959799; SRR959800; SRR959801; SRR959797 |
| floral meristem | SRR959766; SRR959767 |
| vegetative meristem | SRR959773; SRR959772 |
| seed | SRR349645; DRR030759; DRR030758; DRR030760 |
| pollen | SRR3064884; SRR3085237; SRR3084691 |
| plant | SRR563396; DRR015005; DRR015000; DRR015004; DRR014999; DRR015002; DRR015001; DRR014998; DRR015003 |

**Supplemental Table 2. *Sorghum bicolor* samples with organ annotation.**

**Supplemental Methods**

*Supplemetal Method 1. Data source and curation*

The positive dataset of 821 samples for *Arabidopsis thaliana* was manually selected to represent polyA-enriched data of different tissues. The negative dataset consists of 41 runs which represent non-polyA data, as well as samples from other species (Supplemental Table 1). An overview of all *Sorghum bicolor* RNA experiments with their descriptions was downloaded on April 4th 2016 from the SRA (<http://www.ncbi.nlm.nih.gov/sra>, Supplemental table 2, [1]). Using the script get_sra.py, the selected expression data were downloaded as .sra files using the Aspera download client ( <http://downloads.asperasoft.com/en/downloads/2> ). These .sra files were converted into compressed fastq files using the script sra_to_fastq.py, which calls fastq-dump (included in sratoolkit [1]) (options used: --gzip, --skip-technical, --readids, --dumpbase and --split-3). Both scripts are available as helper scripts in the LSTrAP repository.

The TAIR10 release of the *Arabidopsis thaliana* genome [2], including gene annotation, was obtained from [www.arabidopsis.org](http://www.arabidopsis.org). The *Sorghum bicolor* genome (version from Mar 28 2011, [3]) along with its annotation were downloaded from PlantGDB <http://www.plantgdb.org/XGDB/phplib/download.php?GDB=Sb> [4].

*Supplemetal Method 2. PCA analysis of expression data*

The TPM normalized expression matrix (*m* x *n*) containing expression values for 29 448 genes in (*m*) 204 *Sorghum bicolor* samples (*n*) was centered and scaled using the maxscale_abs preprocessing function included in the sklearn toolkit (http://scikit-learn.org , [5]). Values for the first and second principal component of each sample were determined by applying the function sklearnPCA (from the same package) on the matrix. The results were plotted using the python library matplotlib (<http://matplotlib.org/>) in combination with Seaborn (https://stanford.edu/~mwaskom/software/seaborn/). The proportion of overall variability captured by the first and second principal component is shown next to the axes. The script used along with the expression matrix is included in the helper scripts of LSTrAP.

*Supplemetal Method 3. Power law*

LSTraP report co-expressed gene pairs with a PCC > 0.7. Here, we checked the node-degree, the number of genes co-expressed (based on this threshold) for each gene included in the analysis. The number of genes with a specific node degree was counted and plotted using Seaborn. The script used along with the node degrees distribution is included in the helper scripts of LSTrAP.

**References**

1. Leinonen R, Sugawara H, Shumway M, International Nucleotide Sequence Database Collaboration. The sequence read archive. Nucleic Acids Res. [Internet]. Oxford University Press; 2011 [cited 2016 Sep 8];39:D19-21. Available from: http://www.ncbi.nlm.nih.gov/pubmed/21062823

2. The Arabidopsis Genome Initiative. Analysis of the genome sequence of the flowering plant Arabidopsisthaliana. Nature [Internet]. Nature Publishing Group; 2000 [cited 2016 Sep 9];408:796–815. Available from: http://www.nature.com/doifinder/10.1038/35048692

3. Paterson AH, Bowers JE, Bruggmann R, Dubchak I, Grimwood J, Gundlach H, et al. The Sorghum bicolor genome and the diversification of grasses. Nature [Internet]. Nature Publishing Group; 2009 [cited 2016 Sep 9];457:551–6. Available from: http://www.nature.com/doifinder/10.1038/nature07723

4. Duvick J, Fu A, Muppirala U, Sabharwal M, Wilkerson MD, Lawrence CJ, et al. PlantGDB: a resource for comparative plant genomics. Nucleic Acids Res. [Internet]. 2008 [cited 2016 Sep 9];36:D959-65. Available from: http://www.ncbi.nlm.nih.gov/pubmed/18063570

5. Pedregosa F, Varoquaux G, Gramfort A, Michel V, Thirion B, Grisel O, et al. Scikit-learn: Machine Learning in Python. J. Mach. Learn. Res. 2011;12:2825–30.
